# Human-Animal Interaction and the Emergence of SARS-CoV-2

**DOI:** 10.2196/22117

**Published:** 2020-10-07

**Authors:** Asma Hassani, Gulfaraz Khan

**Affiliations:** 1 Department of Medical Microbiology and Immunology College of Medicine and Health Sciences United Arab Emirates University Al Ain United Arab Emirates

**Keywords:** zoonosis, human-animal interface, COVID-19, SARS-CoV-1, outbreak, virus, transmission, pathogen, emergence, reservoir

## Abstract

The COVID-19 pandemic has affected all sectors of society, from health and economics to socialization and travel. The level and extent of this impact are unprecedented. Although the cause of COVID-19 was quickly identified to be a new coronavirus (SARS-CoV-2), the world was poorly prepared for preventing its spread. One important pillar of preparedness is surveillance of the sources of emerging pathogens and responding appropriately to prevent their spread in the human population. The ever-increasing interaction between humans and animals is one leading factor in facilitating the emergence of new pathogens. In this viewpoint, we discuss the possibility of the zoonotic origin of SARS-CoV-2, highlight the importance of understanding human-animal interaction to improve preparedness for future outbreaks, and outline recommendations for prevention.

## Introduction

Currently, the world is battling a pandemic caused by a virus previously undocumented in humans. The new virus, SARS-CoV-2, was identified as the cause of COVID-19. The infection presented with a broad spectrum of symptoms ranging from mild disease to organ failure and death. Similar to severe acute respiratory syndrome coronavirus (SARS-CoV-1) and the Middle East respiratory syndrome coronavirus (MERS-CoV), SARS-CoV-2 is a positive-sense, single-stranded ribonucleic acid (+ssRNA) member of the β-coronavirus genus. However, compared to SARS-CoV-1, SARS-CoV-2 appears to be more efficient in human-to-human transmission [1,2]. The fact that the global spread of the virus outpaced infection control measures has raised concerns about our preparedness to face new viral threats. In this viewpoint, we discuss the potential zoonotic origin of SARS-CoV-2 and revisit the main elements that promote the emergence and spread of new infectious diseases. We also highlight the factors that instigate the rise in new human pathogens and outline recommendations for prevention.

## SARS-CoV-2 and the Possibility of an Animal-to-Human Spillover Event

Despite the uncertainty about where and when SARS-CoV-2 originated, the genome sequencing of isolates from early cases indicate an animal origin, with bats being suggested as the most probable source [3-6]. *In silico* evaluation of a SARS-CoV-2 receptor, angiotensin-converting enzyme 2 (ACE2), indicates that the current pandemic may be caused by a virus with a broad range of hosts, including swine, civets, cats, cows, buffalo, sheep, pigeon, and pangolins [7]. The susceptibility and permissibility of these animals, however, are yet to be tested and proven. One or more domesticated and wild animals have been proposed as an intermediate host for SARS-CoV-2, aiding the spillover to humans [8-11]. Determination of the seroprevalence of SARS-CoV-2 and experimental infection in these animals will help to clarify which, if any, may be involved in the emergence of SARS-CoV-2 [5,7,12]. Moreover, it is also speculated that SARS-CoV-2 could be a result of the recombination of two viruses that circulate in animals living or placed in close proximity [13,14].

If SARS-CoV-2 is the result of a cross-species spillover, the conditions that facilitated the adaptation of SARS-CoV-2 to humans remain unknown [15]. If prejump adaptation occurred in an intermediate host, then SARS-CoV-2 would have undergone genetic refinement in one or more animal species that were spatiotemporally aggregated and in constant contact with humans. The intermediate species is likely to carry an ACE2 receptor that closely clusters with that of humans. If viral adaptation occurred after the spillover event, then continuous passage of the virus from person to person would provide the necessary natural selection opportunities. This would be a realistic assumption given the relatively long incubation (and infectiousness) period, which allows for unnoticeable transmission via a respiratory route. The postadaptation progeny virus can be somewhat genetically distinct from the original virus that made the initial animal-to-human jump [4]. For instance, examining cases of the 2002 severe acute respiratory syndrome (SARS) outbreak revealed that SARS-CoV-1 isolates from early cases (2002) differed from viral isolates obtained from later cases (2003). The viral spike (S) protein involved in binding to the ACE2 receptor showed reduced binding affinity in later viral isolates compared to early isolates [16]. Additionally, a sequence of 29 nucleotides in the C-terminus of the viral genome was deleted in viral isolates from later cases, and this deletion is believed to have occurred through viral adaptation to a human host [17].

## Revisiting the Four Levels of Emerging Infectious Diseases

**Figure 1:**
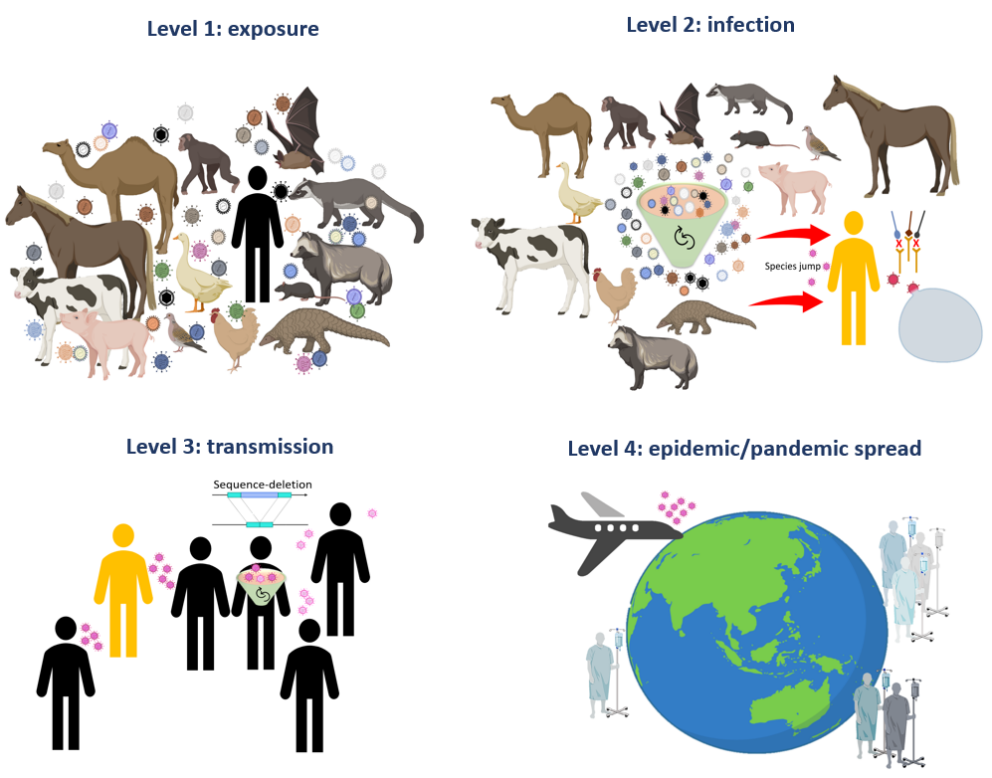
The four-level model of pathogen emergence. Bushmeat production, poultry and livestock industries, and live-animal markets warrant frequent close animal-human and animal-animal contact and increase exposure to pathogens circulating in different species. Frequent exposure (level 1) can turn into a productive infection (level 2), given the necessary viral adaptation has taken place to make humans susceptible. By passaging through humans, the new virus may undergo further adaptation. Pandemic emergence necessitates an efficient human-to-human transmission for sustained propagation. Fast human movement across the globe amplifies the chain of transmission. Efforts should be directed at preventing level 1 (exposure) to significantly decrease the chances of pathogen emergence.

The dramatic rise in human activities in animal farming, the livestock and poultry industries, live-animal markets, and bushmeat has dangerously increased human-animal and animal-animal contact rates [18]. As a result, and combined with careless practices of animal handling, pathogen attack rates have surged substantially. Animals (wildlife and domestic) have gained much attention as candidate reservoirs for numerous, often serious, emerging viral diseases including monkeypox, Ebola, HIV, West Nile virus, rabies, and A/H5N1. The complex link between humans, animals, and emerging pathogens can be simplified in a 4-level model (Figure 1).

At level 1 (or exposure), the human host encounters the “new” pathogen (eg, virus). Handling animals promotes such encounters. This initial encounter can happen independently of the human’s susceptibility to the virus. Susceptibility means that the host has suitable receptor(s) for viral attachment. The number of viruses to which we are exposed is unknown, but given the profound interaction we have with our environment, we can only assume that the exposure is much higher than we can possibly recognize. At level 2 (or infection), the human-virus encounter becomes fruitful. The human host becomes susceptible and part of the viral lifecycle. Over a thousand pathogenic species have successfully achieved this level. In fact, within the last four decades, new human pathogens have been identified at a rate of more than 3 annually. The majority of these pathogens were viruses [19]. This predominance of viruses as causative agents of emerging diseases is partially attributed to enhanced approaches in viral detection and increased viral mutation rates occurring at relatively short spans [20]. Moreover, almost three-quarters of new human viral infections are attributed to viruses of animal origin that crossed the species barrier [21,22]. Indeed, viruses of nonhuman primates and bats are associated with a high relative risk of emergence [23]. At level 3 (or transmission), the new virus can spread from one person to another. This is generally indicated by the basic reproduction number (R_0_), a measure that estimates the number of secondary infections in “naïve” susceptible population [24]. A pathogen with an R_0_ >1 has a better chance of widespread transmission leading to level 4. At level 4 (epidemic/pandemic spread), efficient human-to-human transmission generates sustained propagation. This usually happens without the need for new animal-to-human cross-species transmission events [25].

## Factors That Spark Animal-to-Human Spillover

The cascade of events that led to the emergence of SARS-CoV-2 are likely to be complicated, but there seems to be a belief that humans at some point were exposed to the new virus, probably directly from bats (the reservoir?) [26], or indirectly through an intermediate and/or amplifying host(s) (wild and/or farm animals) [5,27,28]. Indeed, increased aggregation of people, wildlife, and domestic animals in spatiotemporal closeness, and the various ways of handling animal products, could have increased the exposure rate (level 1). In order to understand what could have precipitated this cross-species transmission (level 2), it is critical to identify the reservoir and intermediate hosts. The intermediate host can act as a biological bridge between the reservoir and humans. Despite active infection, the intermediate host may appear healthy or asymptomatic long enough for the virus to jump unnoticed to humans. The intermediate host offers the virus the additional advantage of a suitable environment for rapid evolution priming for the jump from animal species to humans. Postjump changes in the virus also tend to occur in the human host, and the significance of these changes is yet to be unraveled [29].

In addition to intermediate hosts, the reservoir is a key player in pathogen emergence. Fruit bats have been a common source for a broad range of viruses associated with emerging human infections [30,31]. In the beginning of the 1990s, humans who came in close contact with the blood or body fluids of sick, infected horses (intermediate) acquired a new virus known as Hendra virus. Later, fruit bats were found to be the source for this paramyxovirus. Since then, Hendra virus has caused some isolated outbreaks of serious and often fatal infections in horses and humans [32]. Moreover, in a single outbreak in 1997, Menangle virus, another paramyxovirus circulating in bats, spilled over to pigs. Humans working in pig farms were directly exposed to the virus shed in the secretions of infected pigs, or indirectly through contaminated surfaces [33]. Fruit bats appear to harbor viruses with a broad range of hosts; viruses that utilize cellular receptors that are highly conserved across species have better chances of breaching species barrier [34,35]. For example, Nipah virus was a new human pathogen recognized in 1998 in association with pig farming and cases of fatal encephalitis. In addition to humans and pigs, the virus could naturally infect other species including horses, dogs, and cats [36]. Thus, these animals also became hosts for the virus. Although pigs were the intermediate host in the early outbreaks of Nipah in Malaysia and Singapore, more recent outbreaks in India and Bangladesh suggest direct transmission from bats to humans through contact with fruits contaminated with infectious bat fluids (eg, urine, saliva) [37]. Interestingly, numerous RNA viruses circulate in bats. RNA viruses are a predominant cause of newly emerging zoonotic diseases, such as Ebola and SARS-CoV-2. Accelerated rates of mutation and presence of several variants of a given strain provide RNA viruses with unmatched capacity for rapid adaptation in a short time [20].

Collectively, the above mentioned factors explain how activities pertaining to human-animal interaction pose an increasingly high risk of pathogen emergence. We may not have substantial evidence for the involvement of human-animal contact in the emergence of SARS-CoV-2, but the new outbreaks in mink farms in the Netherlands [38,39] certainly highlight how cross-species transmission could occur. Indeed, this possibly bidirectional spread between humans and animals necessitates policies that regulate human-animal contact. Furthermore, the expanding human population has led to more wildlife hunting, bush meat trade and consumption, live animal markets, densely populated livestock and poultry industries, exotic species trading, and rapid animal transportation to meet the growing demand for such products. This has created continuous and intense contact between humans and potential viral reservoirs, vectors, and intermediate hosts. Additionally, with increased human movement at exceptionally accelerated speed, pathogens can be introduced to previously uninfected areas, which are geographically distant from the source of infection, leading to disastrous pandemics across the globe. Given that we do not have a vaccine or immunity against most emerging viral diseases, resources directed at level 1 (ie, exposure) remain our best hope for preventing costly consequences. It is believed that emerging zoonotic diseases involve a complex interaction between multiple anthropogenic factors that modify the structure and dynamics of wildlife [40].

## Recommendations

Recognizing the importance of these factors has led to intensive collaboration between different disciplines, including veterinary medicine, public health, environmental sciences, and social sciences, etc. This holistic approach, known as One Health, has shown promising results in curbing the spread of pathogens between humans and animals and between animals and animals [41]. For instance, the integration of veterinary sciences, epidemiology, and virology has led not only to the identification of the different animal reservoirs for rabies but also to the establishment of surveillance systems for monitoring outbreaks and the development of an effective vaccine [42]. These outcomes led to significant improvements in the control of rabies in both animals and humans. Similarly, a vaccine against Hendra virus helped control infection in horses and hence prevented transmission of the virus to humans [43]. Another example of fruitful multidisciplinary work is monitoring climate change to identify temperatures that favor pathogen transmission. For example, changes in climate, which in turn can affect mosquito movement, have been used in prediction models to monitor the emergence of West Nile virus [44].

Although in principle the One Health approach has been shown to be effective in controlling and preventing infectious diseases outbreaks, in reality, implementing it has proven to be challenging. Factors such as limited investments, lack of collaboration, and suboptimal surveillance systems have all hindered the achievement of optimal outcomes [45]. However, continuous progress in the One Health approach and supporting policies remain an essential shield in the face of future outbreaks [42]. This entails serious efforts to improve and implement:

1. Continuous surveillance of animals and vectors (eg, bats, rats, wild birds, primates, arthropods) for the emergence of new pathogens. This should help predict and prepare for the next pathogen threat and define regions susceptible to outbreaks and tools necessary to interrupt route of transmission. Additionally, further modeling, analyzing, and estimating the risk of emergence can help prepare suitable diagnostic tools for the rapid capture of a new outbreak, and frame essential mitigation protocols [46].
2. International cooperation to establish pathogen sequence libraries and databases updated with biological characterization of new pathogens. This can facilitate efforts in diagnosis, contact tracing, and potential vaccine development.
3. Studying and monitoring human behavior associated with increased risk of emerging diseases such as wildlife trade and bush meat consumption. Social sciences are important to help introduce behavioral modifications that can be beneficial and easily adhered to by communities. This also requires education and increase in public awareness to the dangers of high-risk behaviors.
4. Monitoring alterations in land use, climate change, and air quality, as well as introducing policies to reduce and/or mitigate ecological consequences [42]. Zoonosis is greatly impacted by changes in land use. These changes impact the natural habitat of wild animals and their biodiversity, influence their breeding sites, and determine the exposure rate of humans to pathogens in wild animals. Maintaining the biodiverse pool of wildlife and the natural habitat of wild animals should be a priority when planning landscapes [47].
5. Monitoring and implementing better, safer, and sustainable animal agricultural practices. Overcrowding, mixing of animals, overuse of antibiotics, and methods of transport are some of the drivers of pathogen emergence and re-emergence.

## Conclusion

Humans share a close relationship with animals and their environment. Hence, the risk of emergence of new pathogens that can spill over into the human population cannot be completely eliminated. However, understanding the importance of the human-animal-ecosystem interface and tackling the diverse factors that influence the emergence of new pathogens are essential for better prevention, control, and mitigation. The One Health approach could have a significant positive impact in combatting the emergence of infectious diseases. However, this requires national and international cooperation, sharing of data, funding, implementation of policies and legislation, and political will.

## Abbreviations

+ssRNA: positive-sense, single-stranded RNA
ACE2: angiotensin-converting enzyme 2
MERS-CoV: Middle East respiratory syndrome coronavirus
R_0_: reproduction number
RNA: ribonucleic acid
S: spike
SARS: severe acute respiratory syndrome
SARS-CoV-1: severe acute respiratory syndrome coronavirus
